# A Prognostic Model for Predicting Tumor Mutation Burden and Tumor-Infiltrating Immune Cells in Bladder Urothelial Carcinoma

**DOI:** 10.3389/fgene.2022.708003

**Published:** 2022-02-18

**Authors:** Chengbang Wang, Shaohua Chen, Songheng Li, Hua Mi

**Affiliations:** ^1^ Department of Urology, The First Affiliated Hospital of Guangxi Medical University, Nanning, China; ^2^ Institute of Urology and Nephrology, The First Affiliated Hospital of Guangxi Medical University, Nanning, China; ^3^ Guangxi Collaborative Innovation Center for Genomic and Personalized Medicine, Nanning, China; ^4^ Guangxi Key Laboratory for Genomic and Personalized Medicine, Nanning, China; ^5^ Guangxi Key Laboratory of Colleges and Universities, Nanning, China

**Keywords:** tumor mutational burden, bladder urothelial carcinoma, tumor-infiltrating immune cells, immunotherapy, prognosis

## Abstract

Tremendous progress has been made in development of immunotherapeutic approaches for treatment of bladder urothelial carcinoma (BLCA). However, efficacy and safety of these approaches remain unsatisfactory, necessitating further investigations for identification of indicators for predicting prognosis and efficacy. In this study, we downloaded transcriptomic and clinical data of BLCA patients from The Cancer Genome Atlas (TCGA) database, and identified differentially expressed genes (DEGs) between tumor and normal tissues. We incorporated these DEGs in an intersection analysis with immune-related genes (IRGs) obtained from the Immunology Database and Analysis Portal (ImmPort) database, and identified immune-related DEGs. These genes were subjected to Cox and least absolute shrinkage and selection operator (LASSO) regression analyses, then a prognostic model containing AHNAK, OAS1, NGF, PPY and SCG2 genes was constructed, for prediction of prognosis of BLCA and efficacy of immunotherapy. Finally, we explored the relationship between the prognostic model and tumor mutational burden (TMB), abundance of tumor-infiltrating immune cells (TICs) and immunotherapeutic targets, and found that patients with higher risk score (RS) had poorer prognosis and significantly lower levels of TMB. Patients in the low-RS group exhibited higher numbers of lymphoid cells, whereas those in the high-RS group exhibited higher proportions of myeloid cells. However, patients with high-RS tended to respond better to immunotherapy relative to those in the low-RS group. The constructed prognostic model provides a new tool for predicting prognosis of BLCA patients and efficacy of immunotherapy, offering a feasible option for management of the disease.

## Introduction

Bladder urothelial carcinoma (BLCA) is a common malignancy that negatively affects human health. Approximately 81,400 new cases of bladder cancer, with 17,980 deaths, were reported in the United States in 2020 alone (Siegel et al., 2020). To date, transurethral resection of bladder tumors (TURBT) remains the standard treatment for non-muscle invasive bladder cancer (NMIBC) (Flaig et al., 2020). However, incidence of tumor recurrence after TURBT reportedly ranges from 50 to 70%, with 30% of the patients progressing to muscle-invasive bladder cancer (MIBC) (Kamat et al., 2016). This highly malignant condition, which is characterized by a high rate of postoperative distant metastasis, negatively affects patients’ life quality (Flaig et al., 2020).

The rapid advancement in immunotherapy in recent years has generated a variety of immune checkpoint inhibitors (ICIs) which have been applied for treatment of BLCA since 2016 (Özdemir et al., 2018). In fact, clinical trials have demonstrated their safety and efficacy over second-line therapy (Bellmunt et al., 2017; Powles et al., 2018). Over the past few years, multiple types of ICIs have been approved for clinical treatment of MIBC or metastatic bladder cancer, with encouraging results reported from the clinical trials. Nevertheless, many patients have not benefited from these immunotherapies, as evidenced by low objective remission rates (ORR) of only 15–25% and complete remission rates (CRR) below 10%, as well as incidence of serious treatment-related adverse events (TRAEs) in a subset of patients (Sharma et al., 2016; Balar et al., 2017a; Balar et al., 2017b; Bellmunt et al., 2017). Therefore, there is need to identify novel indicators for predicting efficacy of immunotherapy to enhance efficacy and safety (Havel et al., 2019). Tumor mutational burden (TMB) refers to the total number of substitutions and insertions or deletions per one million bases in exons of genes in a tumor tissue (Gibney et al., 2016). Previous studies have shown that TMBs are correlated with ICIs response rates and survival times of patients with melanoma (Nathanson et al., 2017), as well as breast cancer (Barroso-Sousa et al., 2020), and non-small cell lung cancers (Rizvi et al., 2015; Ettinger et al., 2019). A phase II clinical trial, comprising 310 patients with locally advanced and metastatic BLCA, found that TMB was associated with patients prognosis and could predict treatment responses of atezolizumab (Rosenberg et al., 2016). To date, however, the relationship between TMB and immune response in bladder cancer is still unclear.

In the present study, we performed intersection analysis of differentially expressed genes (DEGs), between tumor tissues of bladder cancer alongside normal controls from The Cancer Genome Atlas (TCGA) database, and immune-related genes (IRGs) from the Immunology Database and Analysis Portal (ImmPort) database, to obtain immune-related DEGs. We performed Cox regression as well as least absolute shrinkage and selection operator (LASSO) regression analyses to screen the identified genes, then constructed a prognostic model for predicting survival times and efficacy of immunotherapy of BLCA. Finally, we explored the relationship between risk score (RS) and abundance of tumor-infiltrating immune cells (TICs), as well as the correlation between RS and immunotherapy targets. Our findings are expected to provide novel insights to guide future development of effective therapies for treatment of BLCA.

## Materials and Methods

### Data Acquisition and Processing

We employed the HTSeq-counts workflow to download transcriptome data for 433 cases, including 414 tumor and 19 normal samples, from TCGA database of BLCA project (https://portal.gdc.cancer.gov/; accessed on 28 March 2021). Corresponding clinical data for the patients, including age at diagnosis, gender, tumor grade, tumor stage, survival time and survival status, were also downloaded using the bcr.xml format in TCGA database through GDC portal (https://portal.gdc.cancer.gov/; accessed on 28 March 2021).

### Screening for Immune-Related DEGs

DEGs between normal and tumor BLCA tissues were identified using “edgeR” and “limma” packages implemented in R software, based on a false discovery rate (FDR) < 0.05 and |log2FC| >1. A volcano plot of the DEGs was then generated using “ggplot2”. IRGs were obtained from ImmPort database (http://www.immport.org/; accessed on 28 March 2021). An intersection analysis of DEGs and IRGs was performed to obtain immune-related DEGs, which were then visualized using the “venn” and “ggplot” packages in R.

### Functional Enrichment Analysis

The “org.Hs.eg.db” package was used to acquire Entrez-IDs for each immune-related DEGs. Gene ontology (GO) and the Kyoto Encyclopedia of Genes and Genomes (KEGG) pathway analyses were then conducted using cutoff criteria of *p* < 0.05 and *q-*value < 0.05. Results were visualized by the “clusterProfiler”, “enrichplot” and “ggplot2” packages in R.

### Construction of a BLCA Prognostic Prediction Model

We used perl scripts to merge clinic characteristics and immune-related DEGs, and generate a matrix showing the survival times, survival status, and levels of gene for each sample. Thereafter, we employed the createDataPartition function in the “caret” package to randomly divide all samples into two groups, namely a training (containing 2/3 of the BLCA samples) and test (containing 1/3 of the BLCA samples) set for cross-validation. Univariate Cox regression analysis was performed using the “survival” package to further obtain immune-related DEGs associated with prognosis of BLCA in the training set, at a threshold *p* < 0.001. We then used the “glmnet” package to perform LASSO regression, and multivariate Cox regression analyses was used to further screen the genes after LASSO regression. The resulting genes were used to construct a BLCA prognostic prediction model. Next, we divided all BLCA samples into high- and low-RS groups, based on median of RS values, then performed Kaplan-Meier survival analysis of both groups and generate time-dependent receiver operating characteristic (ROC) curves to cross-validate the predictive power of the constructed model using the training, test and the combined (containing all of the BLCA samples) sets.

### Identification of Independent Risk Factors Affecting BLCA Prognosis.

The clinical characteristics of all BLCA samples were merged with RS scores to generate a matrix. Univariate Cox regression analysis was performed on clinical characteristics and RS using the “survival” package, followed by multivariate Cox regression analysis to obtain independent risk factors associated with BLCA prognosis. Correlation between clinical characteristics and RS was conducted by the package “limma” and visualized by “ggpubr”.

### Analysis on TICs

We calculated relative abundance of 22 TIC subtypes in tumor samples using the CIBERSORT algorithm (Newman et al., 2015; Chen et al., 2018), then generated bar plots to present the relative proportion of TICs in each sample. The relationship between each TIC subtype and RS was visualized using the “vioplot” package.

### Correlation Between TMB and RS

We downloaded “Masked Somatic Mutation” data, processed by VarScan2, from the TCGA database (https://portal.gdc.cancer.gov/; accessed on 28 March 2021), then applied the perl script to integrate RS and TMB data of BLCA samples. Tumor mutation profiles in high- and low-RS groups were visualized using the “maftools” package, while further analysis and visualization of the high- and low-TMB levels of survival analysis as well as correlation between TMB and RS were performed using “limma”, “survival”, “survminer”, and “ggpubr” packages.

### Correlation Between Immunotherapy and RS

The relationship between RS with immunotherapy targets and effects was analyzed using “limma” and “ggpubr” packages in R.

## Results

### Identification of Immune-Related DEGs

**FIGURE 1:**
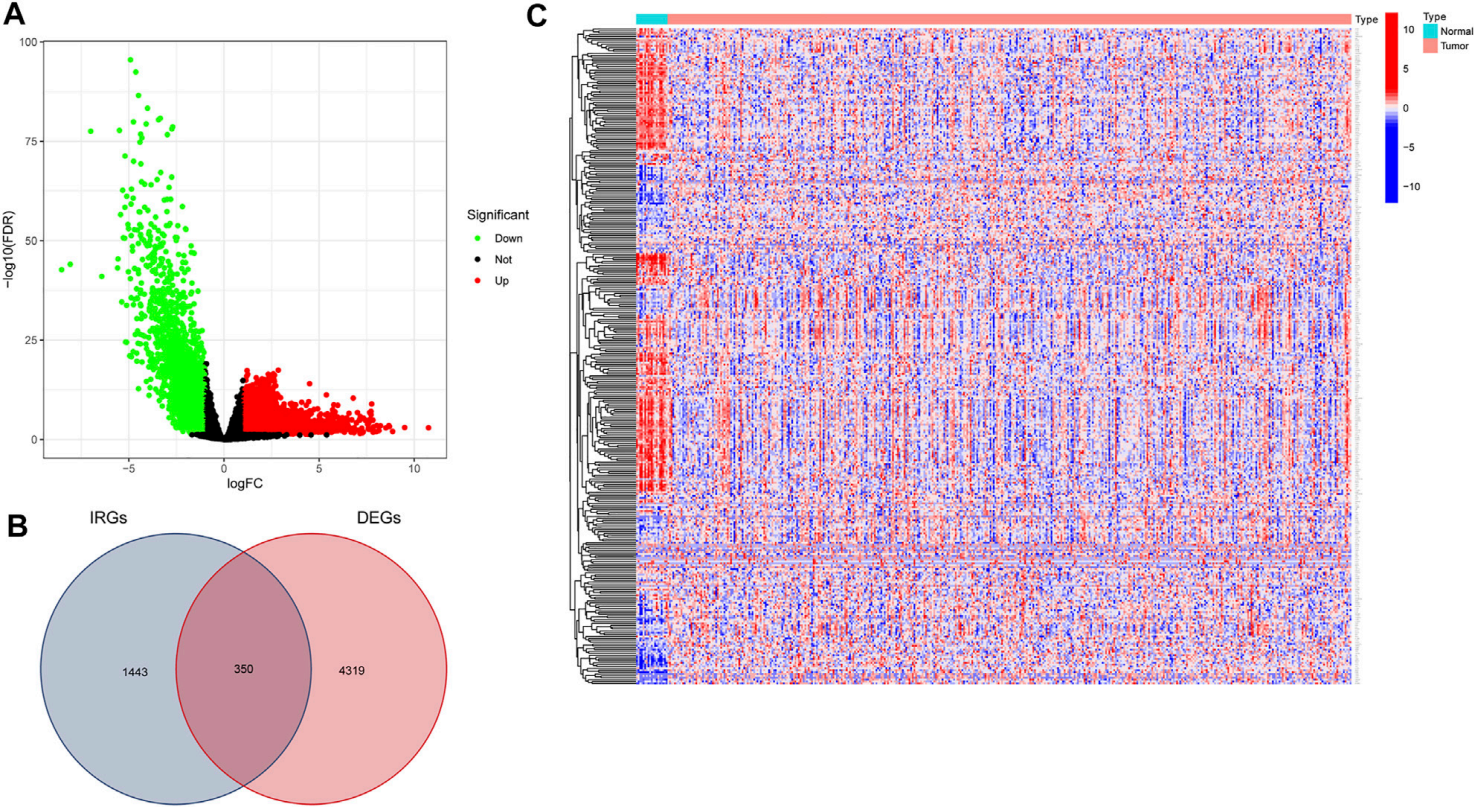
Screening for immune-related differentially expressed genes (DEGs) in bladder urothelial carcinoma (BLCA). **(A)** Volcano plot showing DEGs between tumor and normal tissue based on The Cancer Genome Atlas (TCGA) database of BLCA samples. **(B)** Venn plot showing the intersection analysis of DEGs with immune-related genes (IRGs) from the Immunology Database and Analysis Portal (ImmPort) database. **(C)** Heat map of immune-related DEGs.

**FIGURE 2:**
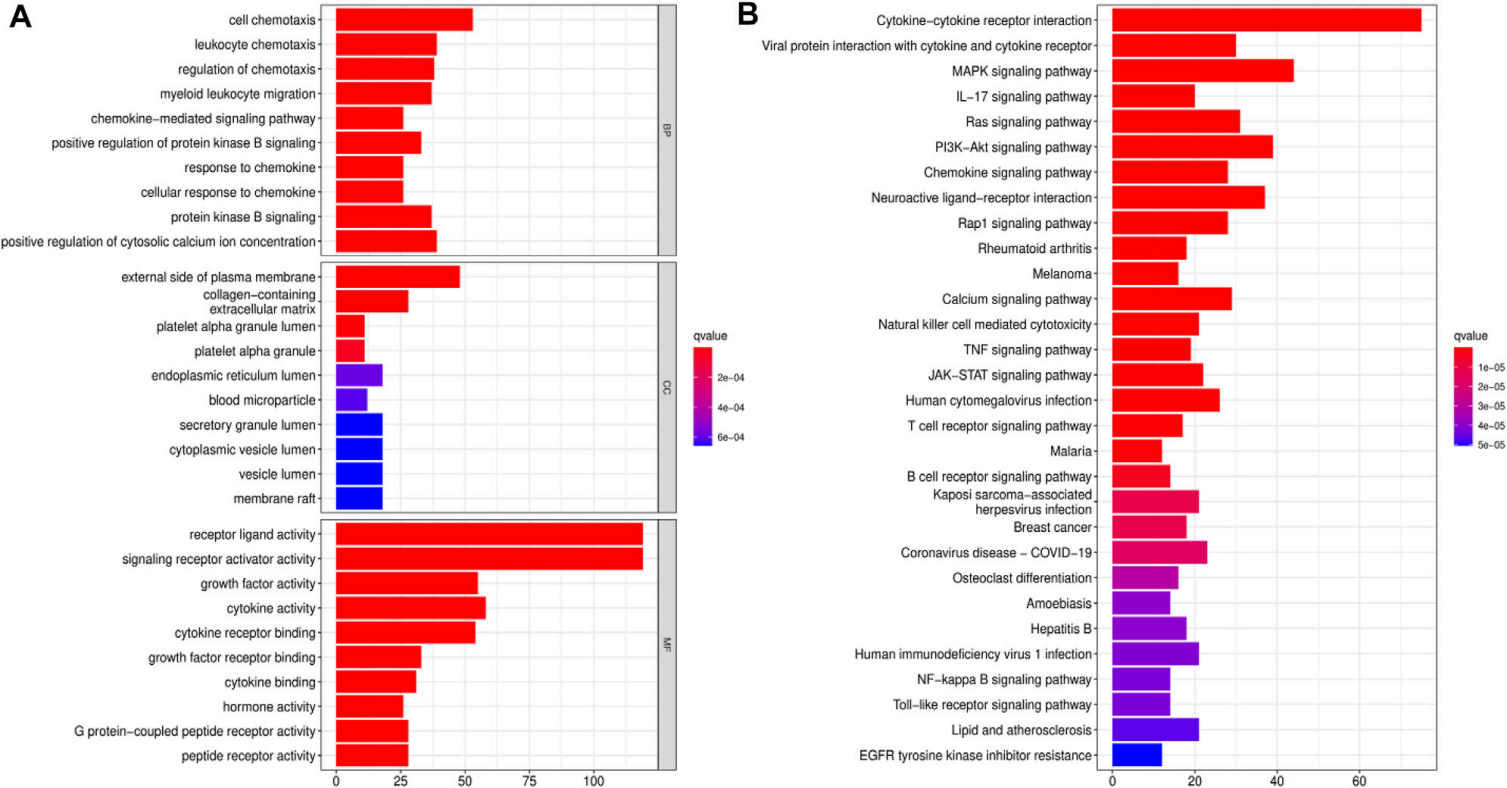
Functional enrichment analyses for immune-related differentially expressed genes (DEGs). **(A)** Gene Ontology (GO) enrichment analysis for immune-related DEGs. **(B)** Kyoto Encyclopedia of Genes and Genomes (KEGG) pathway analysis for immune-related DEGs.

We downloaded data for 433 BLCA samples from the TCGA database, including 414 cancer and 19 normal samples, respectively. Screening for DEGs, based on |log2FC|>1 and FDR<0.05 thresholds, revealed a total of 4,669 DEGs, of which 2,726 and 1943 genes were up-regulated and down-regulated, respectively (Figure 1A) Intersection analysis between the 1793 IRGs from the ImmPort database and DEGs resulted in 350 immune-related DEGs (Figure 1B). Then, we divided the samples into the normal and the tumor type, thereby visualizing relative expression levels of these genes in BLCA patients from TCGA database with heat maps (Figure 1C). GO analysis showed that the immune-related DEGs were mainly enriched in the following pathways: chemokine-related pathway (cellular response to chemokine and chemokine-mediated signaling pathway), biofilm lumen metabolic pathway (external side of plasma membrane and cytoplasmic vesicle lumen) and cytokine ligand receptor activity pathway (growth factor activity and signaling receptor activator activity) (Figure 2A). On the other hand, KEGG pathway enrichment analysis demonstrated that these genes were mainly enriched in cytokine-cytokine receptor interaction and NK cell-mediated cytotoxicity (Figure 2B). Thus, the 350 shared genes obtained by the intersection analysis were uniformly enriched in immune-related activities.

### Construction of a BLCA Prognostic Model

**FIGURE 3:**
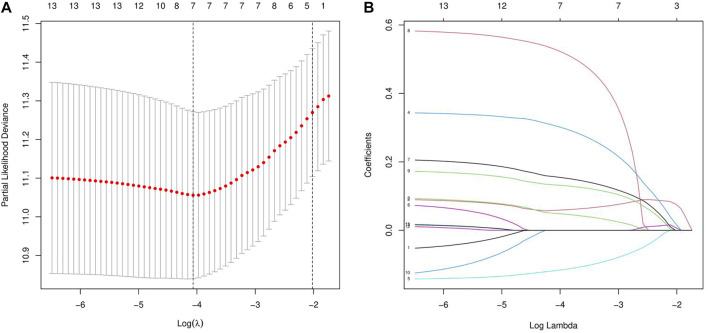
LASSO regression analysis for the screening of immune-related differentially expressed genes (DEGs). **(A, B)** least absolute shrinkage and selection operator (LASSO) regression complexity was controlled by lambda *via* the R package “glmnet”.

**FIGURE 4:**
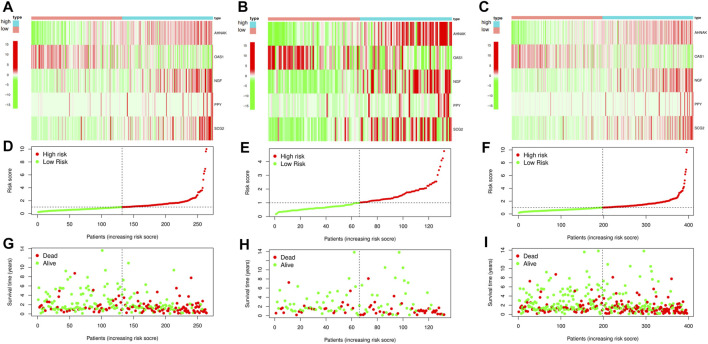
Construction and cross-validation of bladder urothelial carcinoma (BLCA) prognostic model. **(A–C)** The heat map showing the expression levels of the key immune-related differentially expressed genes (DEGs) in the prognostic model for each sample in the training set, test set and combined set, respectively. **(D–F)** Distribution of risk scores (RSs) for each sample in three different datasets. **(G–I)** Distribution of survival states for each sample in three different datasets.

Next, we randomly divided all samples into two groups (ratio of 2:1) for cross-validation, naming training and test sets. As shown in Table 1, no significant differences were observed in the varying clinical characteristics between the training and test sets after random grouping, indicating that they can be used as independent datasets. Univariate Cox regression analysis of 350 immune-related DEGs in the training set revealed 13 genes that were significantly associated with patients’ prognosis (*p* < 0.001) (Table 2). Further screening of the genes using LASSO regression (Figures 3A,B) and multivariate Cox regression (Table 2) analyses revealed 5 immune-related DEGs, which we subsequently used to construct a BLCA prognostic model. We divided the samples into high- and low-RS groups, with values below the median classified into the low-RS group and vice versa. Heat maps for BLCA samples in the training, test and combined sets revealed that OAS1 was downregulated while the other 4 genes were upregulated in the high-RS group (Figures 4A–C), suggesting that individual genes in the prognostic model may play diametrically opposed roles in BLCA. Profiles of RS distribution (Figures 4D–F) and RS-related patient survival status (Figures 4G–I) revealed that an increase in RS significantly increased mortality risk of patients.

**TABLE 1:** Comparison of clinical characteristics between the training and test sets.

Clinical characteristics		Number	Test (%)	Training (%)	*p*-value
Age	≤65	159	46 (34.85)	113 (42.97)	0.149
	>65	236	86 (65.15)	150 (57.03)	
Gender	FEMALE	104	36 (27.27)	68 (25.86)	0.8567
	MALE	291	96 (72.73)	195 (74.14)	
Grade	High Grade	374	128 (96.97)	246 (93.54)	0.4254
	Low Grade	18	4 (3.03)	14 (5.32)	
	unknow	3	0 (0)	3 (1.14)	
Stage	Stage I-II	125	43 (32.58)	82 (31.18)	0.9059
	Stage III-IV	268	89 (67.42)	179 (68.06)	
	unknow	2	0 (0)	2 (0.76)	
T	T1-2	116	38 (28.79)	78 (29.66)	0.9786
	T3-4	247	79 (59.85)	168 (63.88)	
	unknow	32	15 (11.36)	17 (6.46)	
M	M0	189	58 (43.94)	131 (49.81)	0.7877
	M1	10	4 (3.03)	6 (2.28)	
	unknow	196	70 (53.03)	126 (47.91)	
N	N0	228	78 (59.09)	150 (57.03)	0.5096
	N1-3	126	38 (28.79)	88 (33.46)	
	unknow	41	16 (12.12)	25 (9.51)	

**TABLE 2:** Cox regression analysis for screening of immune-related differentially expressed genes (DEGs) affecting the prognosis of bladder urothelial carcinoma (BLCA).

Gene id	Univariate cox analysis		Multivariate cox analysis	
	HR (95%CI)	*p*-value	HR (95%CI)	*p*-value
CXCL12	1.20 (1.08–1.33)	6.90E-04		
LRP1	1.46 (1.23–1.73)	1.50E-05
PDGFRA	1.27 (1.11–1.45)	4.44E-04
AHNAK	1.52 (1.24–1.86)	5.89E-05	1.46 (1.19–1.78)	2.23E-04
OAS1	0.78 (0.68–0.9)	7.96E-04	0.86 (0.74–1.00)	5.54E-02
EDNRA	1.29 (1.11–1.5)	7.05E-04		
NGF	1.54 (1.23–1.92)	1.62E-04	1.23 (0.96–1.57)	1.02E-01
PPY	1.75 (1.32–2.31)	8.52E-05	1.82 (1.37–2.41)	3.24E-05
SCG2	1.26 (1.12–1.43)	1.25E-04	1.21 (1.05–1.39)	8.75E-03
TGFB3	1.23 (1.09–1.39)	7.71E-04		
FGFR1	1.22 (1.09–1.36)	4.98E-04		
NRP2	1.31 (1.15–1.5)	5.34E-05		
PTGER3	1.23 (1.09–1.39)	6.41E-04		

### Cross-Validation of the Prognostic Model

**FIGURE 5:**
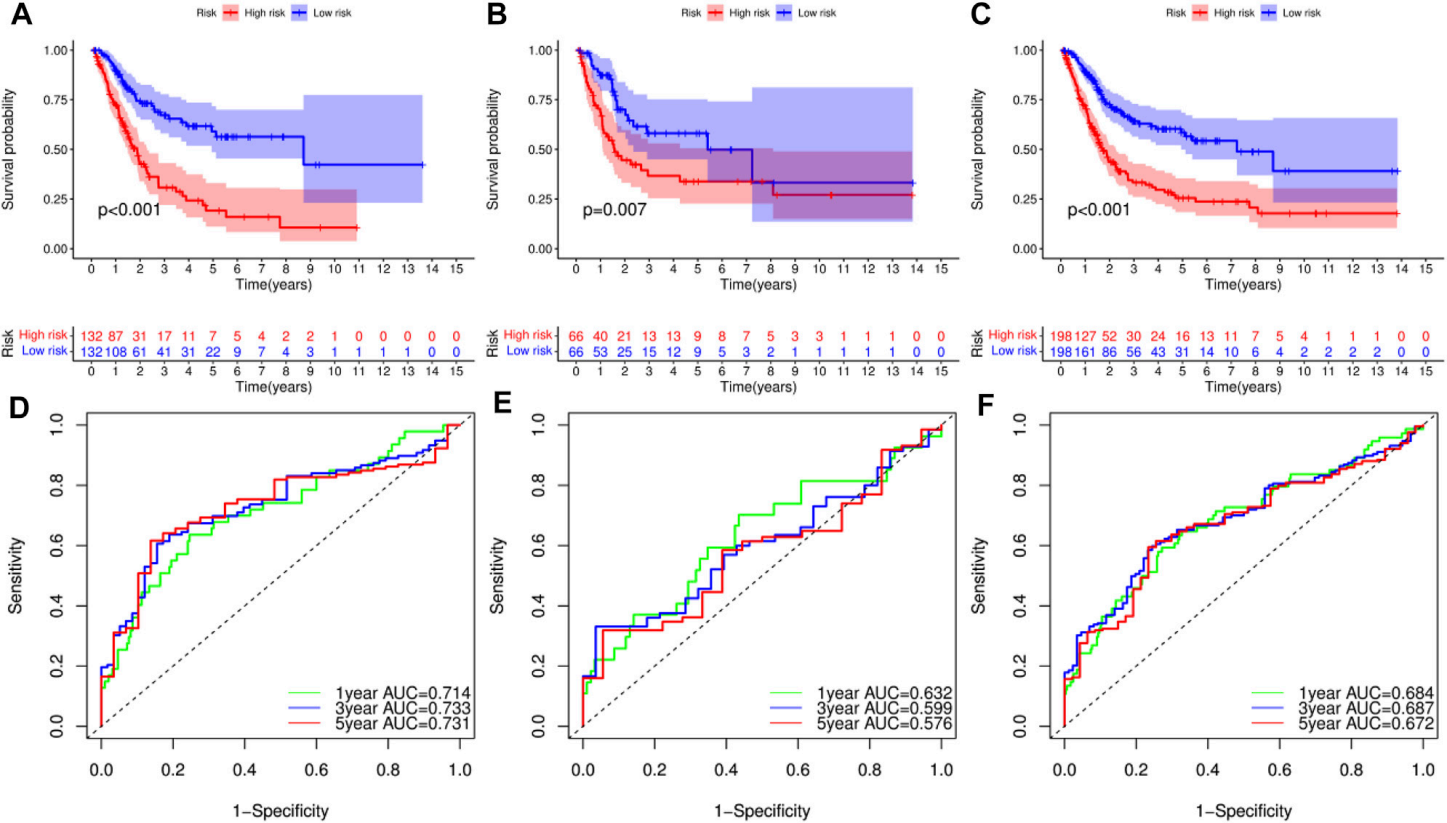
Survival analysis of bladder urothelial carcinoma (BLCA) in different datasets and receiver operating characteristic (ROC) curve analysis for prognostic model. **(A–C)** Survival analysis of BLCA patients with high- and low-risk scores (RSs) in the training set, test set and combined set, respectively. **(D–F)** ROC curve analysis for the prognostic model at 1-, 3- and 5-years in the training set, test set and combined set, respectively.

Survival analysis of the training set, used to validate the constructed model, revealed that patients in the low-RS group had longer overall survival (OS) time than those in the high-RS group (*p* < 0.01, Figure 5A). Survival analysis for the test and combined sets revealed poor prognosis in the high-RS than the low-RS group (*p* < 0.01) (Figures 5B,C). Meanwhile, ROC curves revealed that the prognostic model could efficiently predict clinical outcomes, as evidenced by area under the curve (AUC) values of 0.714, 0.733 and 0.731 for 1-, 3- and 5-years survival, respectively (Figure 5D). Similar results were obtained in the ROC curves analyses for the test and combined sets (Figures 5E,F). Overall, these results indicated that the constructed prognostic model was highly stable and reliable in predicting prognosis of BLCA patients, and guarantees good sensitivity and specificity.

### RS was an Independent Risk Factor for Patient Prognosis

**FIGURE 6:**
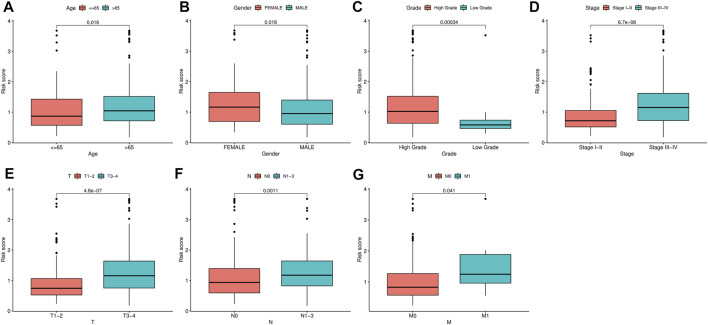
Correlation analysis between clinical characteristics and risk scores (RSs). **(A–G)** The relationship between RS and gender, tumor stage, tumor grade, T classification, and M classification of bladder urothelial carcinoma (BLCA) patients.

To identify the independent risk factors for BCLA development in patients, we used a univariate Cox regression to analyze the relationship between patients’ clinical characteristics and RS in combination with survival time. Results indicated that age, clinical stage and RS were risk factors associated with prognosis of BLCA patients in combined dataset (Table 3). On the other hand, multivariate Cox regression analysis showed that age, clinical stage and RS were independent risk factors associated with prognosis of BLCA (Table 3). Notably, the Cox regression analysis results of the training set and the test set invariably present the similar results, indicating that RS were independent risk factors in these two datasets (Supplementary Tables S1, S2). Thereafter, we correlated RS with clinical characteristics, including age, gender, tumor grade and clinic stage, and found that RS was closely associated with various clinical characteristics (*p* < 0.05) (Figures 6A–G). Meanwhile, tumor grade and tumor stage were positively correlated with RS. These results indicated that RS was related to progression and metastasis of BLCA, implying its potential in predicting prognosis.

**TABLE 3:** Cox regression analysis of clinical characteristics and risk score (RS) affecting patients’ prognosis in combined set.

Variable	Univariate cox analysis		Multivariate cox analysis	
	HR (95%CI)	*p*-value	HR (95%CI)	*p*-value
Age	1.033 (1.017–1.049)	4.6938E-05	1.029 (1.013–1.045)	0.000371
Gender	0.873 (0.628–1.213)	0.41752846		
Grade	2.801 (0.692–11.330)	0.14836518		
Stage	1.744 (1.435–2.119)	2.1573E-08	1.699 (1.396–2.068)	1.25E-07
RiskScore	1.059 (1.039–1.080)	4.808E-09	1.057 (1.036–1.078)	4.08E-08

### TMB Profile

**FIGURE 7:**
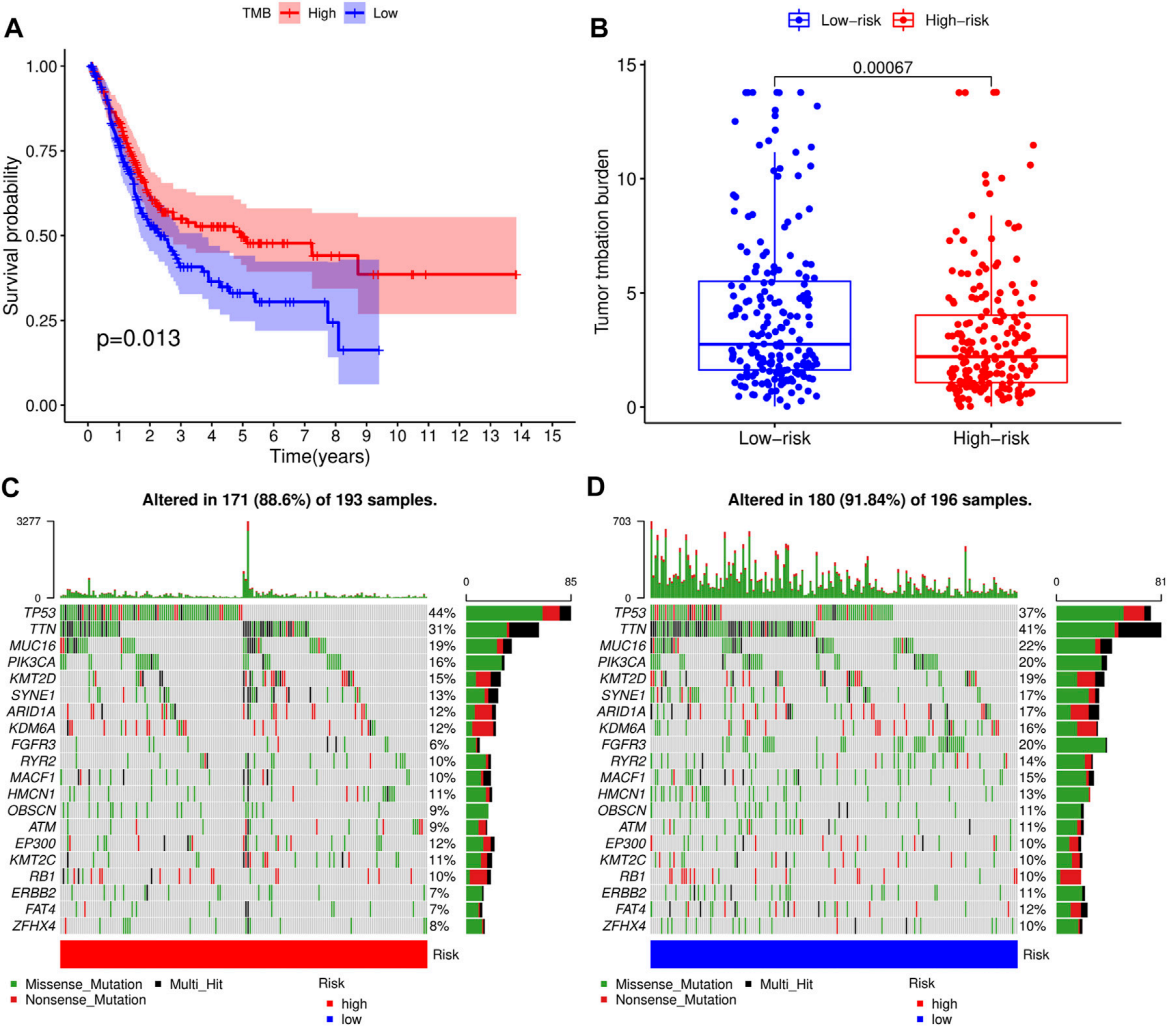
Correlation analysis of tumor mutational load (TMB) and risk scores (RSs). **(A)** Survival analysis for patients with high- and low-TMB. **(B)** Difference of TMB level between high- and low-RS groups. **(C)** Waterfall plot of the top 20 TMB-related genes in the high-RS group. **(D)** Waterfall plot of the top 20 TMB-related genes in the low-RS group.

Next, we analyzed somatic mutation profiles of 405 BLCA patients downloaded from the TCGA database, after dividing them into high- and low-TMB groups based on median TMB value, and combining them with patients’ survival time (Figure 7A). Results showed that patients in high-TMB group had significantly longer survival times than those in the low-TMB group, suggesting that TMB was associated with prognosis of BLCA. Similarly, patients in the low-RS group had significantly higher TMB levels than those in the high-RS group (*p* < 0.01) (Figure 7B), indicating that the prognostic model of BLCA was correlated with TMB. Waterfall plots were used to display detailed mutation information in each sample, with various color annotations used to distinguish between different mutation types (Figures 7C,D).

### TICs and Immunotherapy Analysis

**FIGURE 8:**
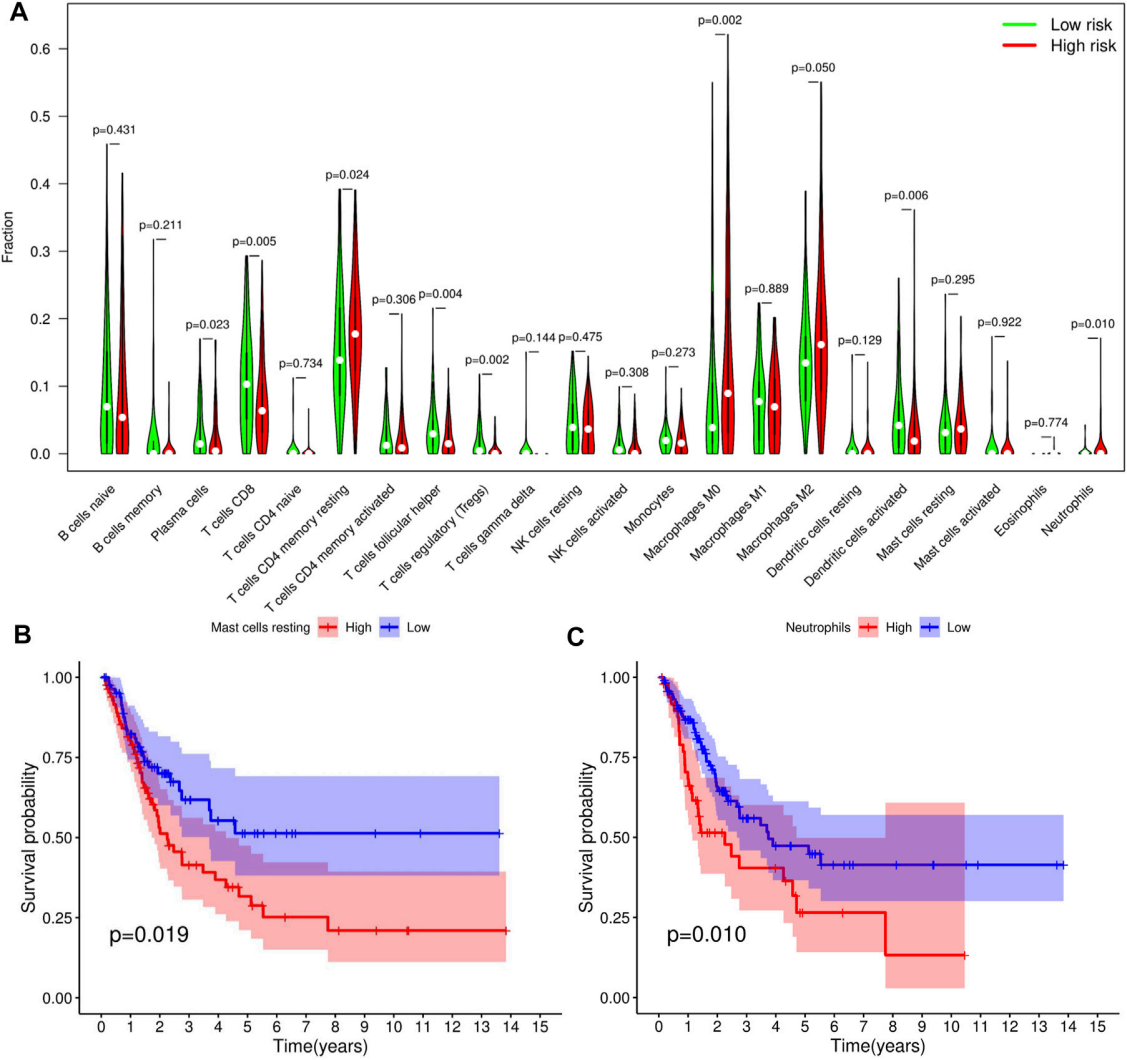
Tumor-infiltrating immune cell (TICs) profile of bladder urothelial carcinoma (BLCA). **(A)** Violin plot showing the difference in the proportion of 22 TIC subtypes in BLCA for the high- and low-risk score (RS) groups. **(B,C)** Two TICs influencing patients’ survival outcome.

**FIGURE 9:**
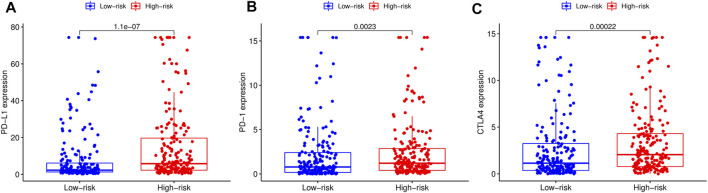
Comparison of the expression levels of immunotherapeutic targets of high- and low-risk score (RS) groups. **(A)** Comparison of programmed cell death ligand 1 (PD-L1) expression levels between high- and low-RS groups. **(B)** Comparison of programmed death 1 (PD-1) expression levels between high- and low-RS groups. **(C)** Comparison of cytotoxic T lymphocyte-associated protein 4 (CTLA4) expression levels between high- and low-RS groups.

We adopted the CIBERSORT algorithm to further analyze relative abundance of various TIC subtypes in tumor samples and revealed that the relationship between prognostic models and TICs. A total of 22 TIC types in tumor samples were compared using the Wilcoxon rank sum test, revealing differences in proportions between the high- and low-RS groups, and the results visualized using violin plot (Figure 8A). Summarily, patients in the low-RS group exhibited significantly higher relative abundance of plasma cells, CD8 T cells, follicular helper T cells, regulatory T cells and activated dendritic cells, while their resting memory CD4 T, macrophages M0 and neutrophils were lower in the high-RS group. We then divided the samples into high- and low-groups, based on the median levels of each TIC, and combined them with survival times. Results indicated that lower proportions of resting mast cells and neutrophils in tumor samples were correlated with longer survival times of patients (*p* < 0.05) (Figures 8B,C). Expression analysis of immunotherapy targets in the high- and low-RS groups revealed upregulation of programmed cell death ligand 1 (PD-L1), programmed death 1 (PD-1), and cytotoxic T lymphocyte-associated protein 4 (CTLA4) in the high-RS group relative to the low-RS group (Figures 9A–C), indicating that patients in high-RS group were more likely to benefit from immunotherapy.

## Discussion

BLCA, the ninth most common cancer in the world that poses a major challenge to global public health (Cumberbatch et al., 2018), is characterized by high aggressiveness and a high recurrence rate. Traditional pathological cystoscopy and urine cytology have been used as for clinical diagnosis of BLCA (Tran et al., 2021). To date, cisplatin-based chemotherapy remains the first-line treatment therapy for locally advanced or metastatic BLCA (von der Maase et al., 2005). However, the strategy is not effective since it requires patients to have renal function reserve and good physical condition, and has also been associated with various adverse effects (Chen et al., 2021b). In recent years, immunotherapy has rapidly developed, giving rise to a variety of ICIs that have consequently been used for treatment of advanced BLCA (Chism, 2017; Siefker-Radtke and Curti, 2018). Although previous clinical studies have demonstrated the strategy’s safety and efficacy, nothing is known regarding indicators for assessment of ICI efficacy. Despite researchers identifying various markers associated with immunotherapy response, such as TMB, TICs, and immune gene signatures, limitations and discrepancies among studies have constrained their application (Gibney et al., 2016; Chan et al., 2019; Samstein et al., 2019).

In the present study, we screened BLCA samples and identified immune-related DEGs, and used them to construct a novel prognostic model. We systematically investigated the relationship between our prognostic model with TMB, TICs and immunotherapeutic targets, and validated its reliability in a test set. The model integrated five immune-related DEGs, namely AHNAK, OAS1, NGF, PPY and SCG2, of which AHNAK, OAS1 and NGF were cell cycle-related. Specifically, AHNAK enhances transcriptional activity of receptor-regulated Smads (R-Smads), causing cell cycle arrest, and participates in cell growth regulation by potentiating transforming growth factor β (TGFβ) signaling (Lee et al., 2014). Moreover, it functions as a tumor suppressor and has also been shown to play an adjuvant role during diagnosis of BLCA (Lee et al., 2018). The 2′-5′ oligoadenylate synthetases (OAS) are interferon-inducible enzymes that recognize viral double-stranded RNA (Kristiansen et al., 2011). Previous studies have shown that OAS1 could suppress accumulation of excess Poly (ADP-ribose) (PAR) in response to DNA damage, thereby inhibiting programmed cell death due to energy depletion and/or activation of PAR (Kondratova et al., 2020). Moreover, Qu and colleagues once proposed a IRG-based prognostic index containing AHNAK and OAS1 that could assess immune status and prognosis with BLCA (Qu et al., 2021). Besides, NGF is a member of the neurotrophic factor family that protects peripheral nerve cells (Rocco et al., 2018). Previous studies have shown that NGF ameliorates the inhibitory effect of the neurotrophin (NTR) receptor on cell-cycle protein expression in cancer cells of BLCA (Khwaja and Djakiew, 2003). However, the roles played by PPY and SCG2 in BLCA remain unclear, necessitating further studies. In the present study, we analyzed the relationship between clinical characteristics of BLCA and RS, and found that patients in low-RS group had better survival outcomes than those in the high-RS group. Notably, older and male patients exhibited significantly higher levels of RS than younger and female ones, in keeping with the findings of Shariat et al. who demonstrated that BLCA was a highly prevalent in the middle-aged and elderly population, with a median age of diagnosis around 70 (Shariat et al., 2010).

Since TICs have been shown to play a crucial role in cancer development and metastasis (Chen et al., 2021a), we further investigated the relationship between TICs and RS. Our results indicated that patients in the low-RS group predominantly exhibited higher numbers of lymphoid cells, whereas those in the high-RS group had higher proportions of myeloid cells. Previous studies have shown that high neutrophil/lymphocyte ratio (NLR) are associated with poor prognosis of several malignancies (Templeton et al., 2014). Survival analysis revealed a significant correlation between lower levels of neutrophils and resting mast cell infiltration with better survival outcomes. Notably, neutrophils play a double-edged role in bladder cancer, stimulating anti-tumor immune responses by releasing IFN-γ (Xiang et al., 2020) or inducing inflammation and production of growth factors and neutrophil elastase to favor tumor growth (Galdiero et al., 2018). Application of ICIs in recent years has generated encouraging efficacy in management of various solid tumors, including bladder cancer (Nadal and Bellmunt, 2019). However, this therapy has been found to have clinical benefits in less than half of patients with advanced BLCA (Crispen and Kusmartsev, 2020). In the present study, we explored the relationship between RS and immunotherapeutic targets, and found significant upregulation of PD-L1, PD1 and CTLA4 in patients in the high-RS relative to those in the low-RS group. These results suggested that better outcomes may be achieved with ICIs in patients with high-RS.

## Conclusion

In summary, we constructed and validated a reliable model for predicting prognosis of BLCA patients, based on transcriptomic and clinical data obtained from public databases. In addition, we elaborated the correlation between the prognostic model and TMB and TICs using correlation analysis. The constructed model provides a new assessment tool for predicting prognosis of BLCA patients and efficacy of immunotherapy, therefor offering an alternative for management of the disease.

## Data Availability

The original contributions presented in the study are included in the article/Supplementary Material, further inquiries can be directed to the corresponding author.
