# Supplementary material for: A Prognostic Model for Predicting Tumor Mutation Burden and Tumor-Infiltrating Immune Cells in Bladder Urothelial Carcinoma
Source: Front Genet. 2022 Feb 18;13:708003. doi: 10.3389/fgene.2022.708003 (PMC8896886; doi:10.3389/fgene.2022.708003)
Supplement: Supplementary file 1 [file Table1.docx]

Supplementary Table S1

Cox regression analysis of clinical characteristics and risk score (RS) affecting patients’ prognosis in training set.

| Variable | Univariate Cox analysis | | Multivariate Cox analysis | |
| --- | --- | --- | --- | --- |
|  | HR（95%CI） | P-value | HR（95%CI） | P-value |
| Age | 1.033（1.013-1.053） | 0.001 | 1.029（1.009-1.049） | 0.005 |
| Gender | 0.900（0.603-1.343） | 0.606 |  |  |
| Grade | 2.316（0.570-9.415） | 0.241 |  |  |
| Stage | 1.709（1.352-2.159） | <0.001 | 1.670（1.318-2.117） | <0.001 |
| RiskScore | 1.065（1.040-1.090） | <0.001 | 1.063（1.038-1.088） | <0.001 |
