# Supplementary material for: A Prognostic Model for Predicting Tumor Mutation Burden and Tumor-Infiltrating Immune Cells in Bladder Urothelial Carcinoma
Source: Front Genet. 2022 Feb 18;13:708003. doi: 10.3389/fgene.2022.708003 (PMC8896886; doi:10.3389/fgene.2022.708003)
Supplement: Supplementary file 3 [file Table2.docx]

Supplementary Table S2

Cox regression analysis of clinical characteristics and risk score (RS) affecting patients’ prognosis in test set.

| Variable | Univariate Cox analysis | | Multivariate Cox analysis | |
| --- | --- | --- | --- | --- |
|  | HR（95%CI） | P-value | HR（95%CI） | P-value |
| Age | 1.033（1.005-1.061） | 0.020 | 1.029（1.001-1.055） | 0.040 |
| Gender | 0.856（0.474-1.547） | 0.607 |  |  |
| Stage | 1.833（1.291-2.602） | 0.001 | 1.498（1.030-2.179） | 0.035 |
| RiskScore | 2.029（1.483-2.776） | <0.001 | 1.761（1.264-2.453） | 0.001 |
